# Unraveling the spatial and signaling dynamics and splicing kinetics of immune infiltration in osteoarthritis synovium

**DOI:** 10.3389/fimmu.2025.1521038

**Published:** 2025-03-13

**Authors:** Chuan Wang, Zevar Zeng, Tao Wang, Zhihong Xie, Jian Zhang, Wentao Dong, Fei Zhang, Wuxun Peng

**Affiliations:** ^1^ Emergency Surgery, Affiliated Hospital of Guizhou Medical University, Guiyang, China; ^2^ School of Life Sciences, Sun-Yat-sen University, Guangzhou, China

**Keywords:** osteoarthritis synovium, immune infiltration, spatial and signaling dynamics, splicing kinetics, spatial transition tensor

## Abstract

**Introduction:**

Osteoarthritis (OA), a debilitating joint disorder characterized by synovial inflammation and immune myeloid cell infiltration, currently lacks a comprehensive spatial and transcriptional atlas. This study investigates the spatial dynamics, splicing kinetics, and signaling pathways that drive immune infiltration in OA synovium.

**Methods:**

We integrated single-cell RNA sequencing (scRNA-seq) data from 8 OA and 4 healthy synovial samples with spatial transcriptomics using Spatrio. Spatial transition tensor (STT) analysis decoded multistable spatial homeostasis, while splicing kinetics and non-negative matrix factorization (NMF) identified gene modules. CellPhoneDB and pyLIGER mapped ligand-receptor interactions and transcriptional networks.

**Results:**

Re-annotation of scRNA-seq data resolved synovial cells into 27 subclasses. Spatial analysis revealed OA-specific attractors (8 in OA vs. 6 in healthy samples), including immune myeloid (Attractor3) and lymphoid infiltration (Attractor4). Key genes OLR1 (myeloid homeostasis) and CD69 (T-cell activation) exhibited dysregulated splicing kinetics, driving inflammatory pathways. Myeloid-specific transcription factors (SPI1, MAF, NFKB1) and lymphoid-associated BCL11B were identified as regulators. Computational drug prediction nominated ZILEUTON as a potential inhibitor of ALXN5 to mitigate myeloid infiltration.

**Discussion:**

This study delineates the spatial and transcriptional landscape of OA synovium, linking immune cell dynamics to localized inflammation. The identification of OLR1 and CD69 as spatial homeostasis drivers, alongside dysregulated signaling networks, offers novel therapeutic targets. These findings advance strategies to modulate immune infiltration and restore synovial homeostasis in OA.

## Introduction

Osteoarthritis (OA) is a prevalent and debilitating joint disorder characterized by the progressive degradation of articular cartilage, subchondral bone changes, and synovial inflammation, ultimately leading to pain and impaired mobility (1, 2). The synovial membrane, which lines the joint cavity, plays a pivotal role in maintaining joint homeostasis by producing synovial fluid that lubricates and nourishes the cartilage (3–5). In OA, the synovial membrane undergoes significant pathological changes, including hyperplasia and infiltration by immune cells, particularly myeloid cells such as macrophages and monocytes (6–8). These myeloid cells are key mediators of inflammation and tissue remodeling, contributing to the chronic inflammatory environment observed in OA joints (9–11).

Recent advances in single-cell RNA sequencing (scRNA-seq) have revolutionized our understanding of tissue heterogeneity and cellular interactions by enabling high-resolution mapping of cellular states and their spatial organization within tissues (12–14). However, spatial transcriptomics sequencing data for osteoarthritis are lacking. Existing studies have only explored the mechanisms of osteoarthritis at the cellular level (15). The etiology of osteoarthritis is a spatially regulated process and revealing perturbations in regions or cells spatially involved in osteoarthritis can help us optimize osteoarthritis medication and deepen our understanding of the mechanism (16, 17).

**Figure 1:**
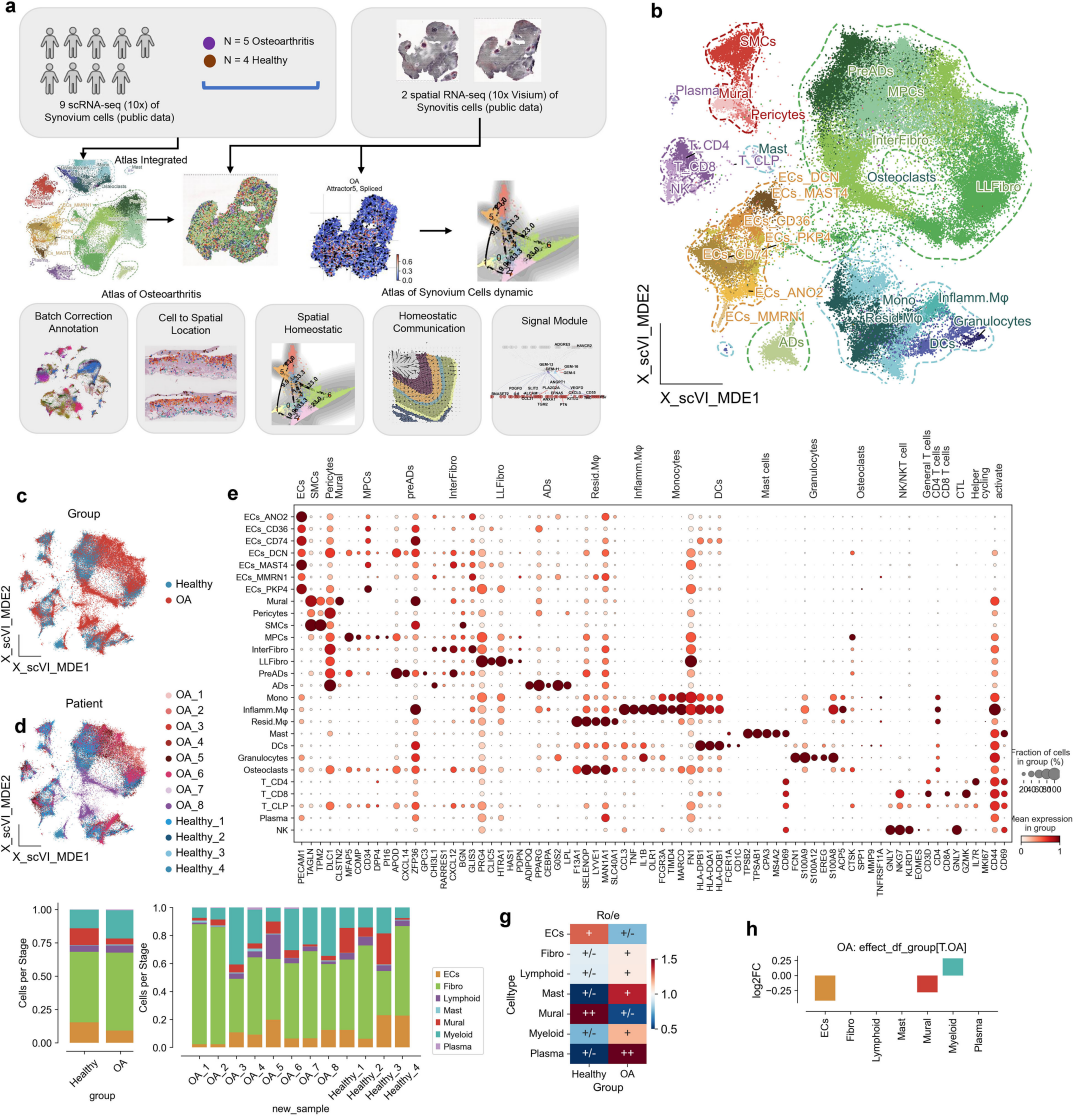
Cellular and molecular landscape of osteoarthritis (OA) and healthy synovium. **(a)** Schematic overview of the study design. The analysis is based on 8 OA cases and 4 healthy controls, leveraging single-cell RNA sequencing data. Synovium from synovitis serves as a reference for single-cell localization in OA. RNA splicing kinetics and spatial transition tensors are used to investigate spatial homeostasis, while CellPhoneDB and flowsig analyses reveal disruptions in spatial signaling. **(b)** UMAP visualization of synovial tissue single-cell RNA sequencing data, organized by cell type: endothelial cells (ECs), fibroblasts (Fibro), lymphoid cells (Lymphoid), mast cells (Mast), mural cells (Mural), myeloid cells (Myeloid), and plasma cells (Plasma). **(c)** UMAP plot highlighting OA (red) and healthy (blue) conditions, showcasing the distribution of cells by condition. **(d)** UMAP plot distinguishing cells according to patient ID and condition, facilitating patient-specific analysis. **(e)** Dot plot illustrating expression levels of marker genes across cell types. Dot size reflects the fraction of cells expressing each gene, and color intensity represents average expression. **(f)** Bar plot showing the cell type proportions in OA and healthy samples, highlighting differences in cellular composition. **(g)** Heatmap of cell type enrichment in OA versus healthy synovium, with the color scale representing log fold changes; positive values indicate higher abundance in OA. **(h)** Bar plot depicting log2 fold change (log2FC) in cell type abundance in OA compared to healthy synovium, calculated by scCODA.

In this study, we first re-annotated the existing single-cell data on osteoarthritis, adjusting the subpopulation resolution from 7 major classes to 27 subclasses. The finer mapping of osteoarthritis cells helps us to refine the cellular mechanisms of OA. To obtain the spatial location of osteoarthritis cells in the synovium, we used the rheumatoid arthritis (RA) synovial membrane spatial profile as a reference atlas to project the single cells spatially using spatrio. We also computed the splicing kinetics of each cell with OA, and the local splicing kinetics of the projected atlas were divided into spatial attractors. Finally, we constructed gene modules on the spatial atlas using non-negative matrix (NMF) decomposition, and we investigated the spatial signaling flow signal with transcript factor in normal synovium and synovium in osteoarthritis based on the ligand-receptor database CellPhoneDB (**Figure 1a**).

Specifically, we identify significant spatial attractor in the synovial membrane architecture and extensive infiltration of myeloid cells in OA compared to healthy controls. Through detailed transcriptional profiling, we reveal key differentially expressed genes (DEGs) and transcription factors (TFs) driving the inflammatory and remodeling processes in OA. We then prediceted the potential drug ZILEUTON to target ALXN5 of the exceptionally myeloid and remove the osteoarthritis-specific myeloid cells. Our study reveals, for the first time, the kinetic characteristics and inflammatory mechanisms of lymphatic infiltration on the spatial surface of osteoarthritis.

## Result

### Single-cell atlas reveals cellular heterogeneity in osteoarthritis synovium

To elucidate the cellular landscape of the synovial membrane in osteoarthritis (OA), we first reanalyzed publicly available single-cell RNA sequencing (scRNA-seq) data from the Gene Expression Omnibus (GEO) database included 8 OA samples and 4 healthy samples. Then we used Spatrio to spatially locate the scRNA profile onto rheumatoid arthritis (RA) synovial membrane spatial profile (**Figure 1a**, **Table 1**). Our analysis aimed to characterize the various cell populations and their transcriptional states, as well as to identify the cellular disruptions associated with OA both cellular and spatial level.

**Table 1:** Information of healthy donor and OA patients whose biospecimens were used for scRNA-seq and snRNA-seq experiments.

Dataset	Sample Type	Sample ID	Age	Gender	BMI (kg/m²)	Joint	Disease Stage/Type
GSE216651	OA	Experiment 1	82	F	22.8	Knee (R)	Knee OA
GSE216651	OA	Experiment 2	75	F	21.7	Knee (R)	Knee OA
GSE216651	OA	Experiment 3	64	F	33.7	Knee (L)	Knee OA
GSE216651	Healthy	Experiment 4	33	M	22.5	Knee (R)	Healthy Knee Joint
GSE216651	Healthy	Experiment 5	21	F	21.2	Knee (R)	Healthy Knee Joint
GSE216651	Healthy	Experiment 6	37	M	21.2	Knee (R)	Healthy Knee Joint
GSE216651	OA	snRNA-seq 1	78	F	23.1	Knee (L)	Knee OA
GSE216651	OA	snRNA-seq 2	79	F	22.5	Knee (L)	Knee OA
GSE216651	Healthy	snRNA-seq 3	37	M	21.2	Knee (R)	Healthy Knee Joint
GSE152805	OA	GSE152805_OA_1	-	F	-	Knee	Medial compartment dominant knee OA, undergoing total knee replacement (inferred)
GSE152805	OA	GSE152805_OA_2	-	F	-	Knee	Medial compartment dominant knee OA, undergoing total knee replacement (inferred)
GSE152805	OA	GSE152805_OA_3	-	M	-	Knee	Medial compartment dominant knee OA, undergoing total knee replacement (inferred)

Using a combination of dimensionality reduction techniques, we generated a UMAP visualization that revealed distinct clusters representing various cell types included 78,260 cells within the synovial membrane, including endothelial cells (ECs, 9,262), fibroblasts (Fibro, 43,889), lymphoid cells (Lymphoid, 3,999), mast cells (Mast, 76), mural cells (Mural, 6,177), myeloid cells (Myeloid, 14,226), and plasma cells (Plasma, 239) (**Figure 1b**; **Supplementary Figures 1a-c**). These clusters were annotated based on the expression of canonical marker genes, as illustrated in the accompanying dot plot (**Figure 1e**; **Supplementary Figure 1d**). The UMAP plot further highlighted the differential distribution of cells between OA and healthy synovium, with OA groups showing a distinct clustering pattern compared to healthy controls (**Figure 1c**). At the patient level, the variability in cell distribution was evident, reflecting the heterogeneity of the disease (**Figure 1d**).

We first compared the changes in cell proportions between OA and healthy synovium using the Ro/e metric, revealing that endothelial cells (ECs) and mural cells were less abundant in OA, while mast cells, myeloid cells, and plasma cells were more abundant (**Figure 1g**). To account for potential sample-specific biases, we employed scCODA, a Bayesian statistical method, to rigorously assess proportional differences. This analysis confirmed a significant increase in myeloid cell proportions in OA and a decrease in mural cell and EC proportions (**Figure 1h**).

Overall, our comprehensive single-cell atlas of the synovial membrane in OA reveals significant alterations in cellular composition and transcriptional dynamics, particularly the marked infiltration and enrichment of myeloid cells. These findings underscore the importance of myeloid cells in the inflammatory milieu of OA and provide a valuable resource for understanding the cellular mechanisms underlying OA pathogenesis.

### Spatial domains define distinct microenvironments in osteoarthritis synovium

**Figure 2:**
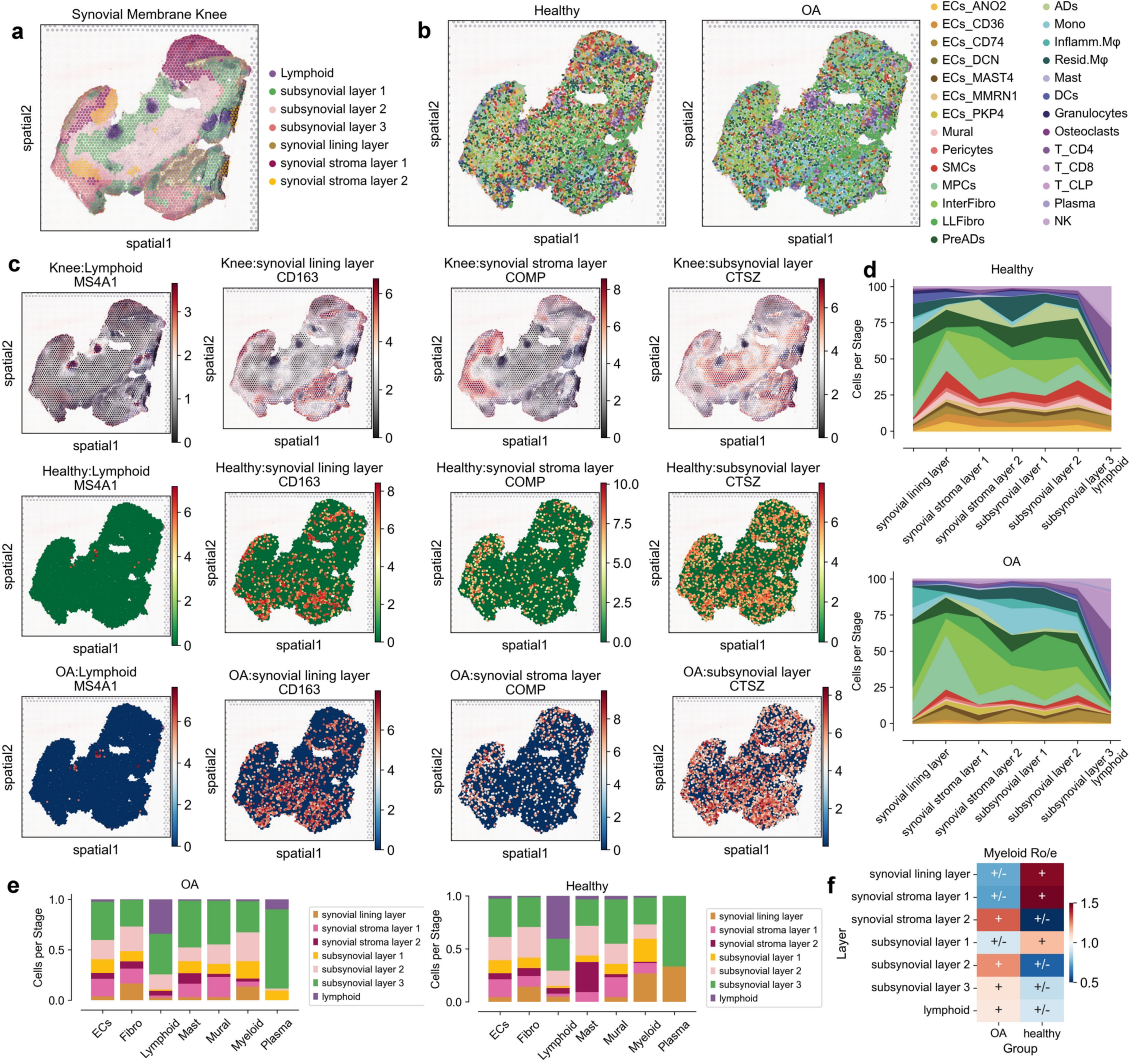
Spatial distribution and cellular composition of synovial membrane in healthy and osteoarthritis (OA) conditions. **(a)** Spatial map highlighting anatomical layers of the synovial membrane knee, including lymphoid, sub-synovial layers 1-3, synovial lining layer, and synovial stroma layers 1-2. **(b)** Spatial distribution of cell types in healthy and OA synovial tissues. Cells are color-coded by type, including endothelial cells (ECs), fibroblasts (Fibro), lymphoid cells (Lymphoid), mast cells (Mast), mural cells (Mural), myeloid cells (Myeloid), and plasma cells (Plasma). **(c)** Spatial expression patterns of marker genes MS4A1, CD163, COMP, and CTSZ across different layers in both healthy and OA conditions. **(d)** Proportion of each cell type across anatomical layers in healthy and OA synovial tissues, shown as area plots with distinctive colors for each layer. **(e)** Bar plots indicating the relative abundance of each cell type across different anatomical layers for healthy and OA synovial tissues, with cell types color-coded and proportionally represented. **(f)** Heatmap showing relative enrichment (Ro/e) of Myeloid cells across layers in OA and healthy groups, with color scale depicting log fold changes; positive values imply higher abundance in OA.

To uncover the spatial organization and domain-specific cellular distribution in the synovial membrane of osteoarthritis (OA) patients, we leveraged spatial transcriptomics data alongside single-cell RNA sequencing (scRNA-seq) data. Our analysis involved the integration of spatial and single-cell datasets to map the spatial locations of various cell types identified in our single-cell atlas. Initially, we conducted spatial clustering of the synovial membrane using STAGATE, which allowed us to define distinct spatial domains within the tissue. These domains were annotated based on hematoxylin and eosin (HE) staining and the expression of region-specific markers, revealing the anatomical structure of the synovial membrane, including lymphoid aggregates, sub-synovial layers, synovial lining layers, and synovial stroma layers (**Figure 2a**) (18). To assign spatial coordinates to the single cells, we utilized Spatrio to locate the single-cell profile in synovial membrane spatial slice. This method enabled us to infer the spatial positions of 10,000 randomly selected cells from both OA and healthy groups. The resulting spatial maps displayed a clear distinction in the spatial distribution of cell types between OA and healthy synovium (**Figure 2b**; **Supplementary Figures 2a, b**). To verify the distribution of our cells, we also performed spatial location matching on another spatial slice of the synovium and found that the cells were consistent with the spatial locations (**Supplementary Figures 2c, d**, **3a, b**).

By examining the expression of key marker genes within these spatial domains, we observed distinct spatial patterns of gene expression in OA compared to healthy synovium. For instance, the expression of MS4A1, CD163, COMP, and CTSZ highlighted the differential localization of lymphoid and myeloid cells, as well as fibroblasts, within the synovial membrane (**Figure 2c**; **Supplementary Figure 3c**). These spatial expression patterns were consistent with the histological features observed in the HE-stained sections. Notably, myeloid cells and plasma cells were enriched in specific spatial domains in OA, suggesting localized inflammatory responses (**Figure 2d**; **Supplementary Figure 3d**). To validate these findings, we analyzed the cell type proportions within each spatial domain. This analysis confirmed the significant decreased of myeloid cells in the synovial lining layer and increased in sub-synovial layers 2-3 in OA, while other cell types showed variable distributions across the spatial domains (**Figures 2e, f**; **Supplementary Figure 3e**).

### Spatial attractors reveal disrupted immune homeostasis in osteoarthritis synovium

**Figure 3:**
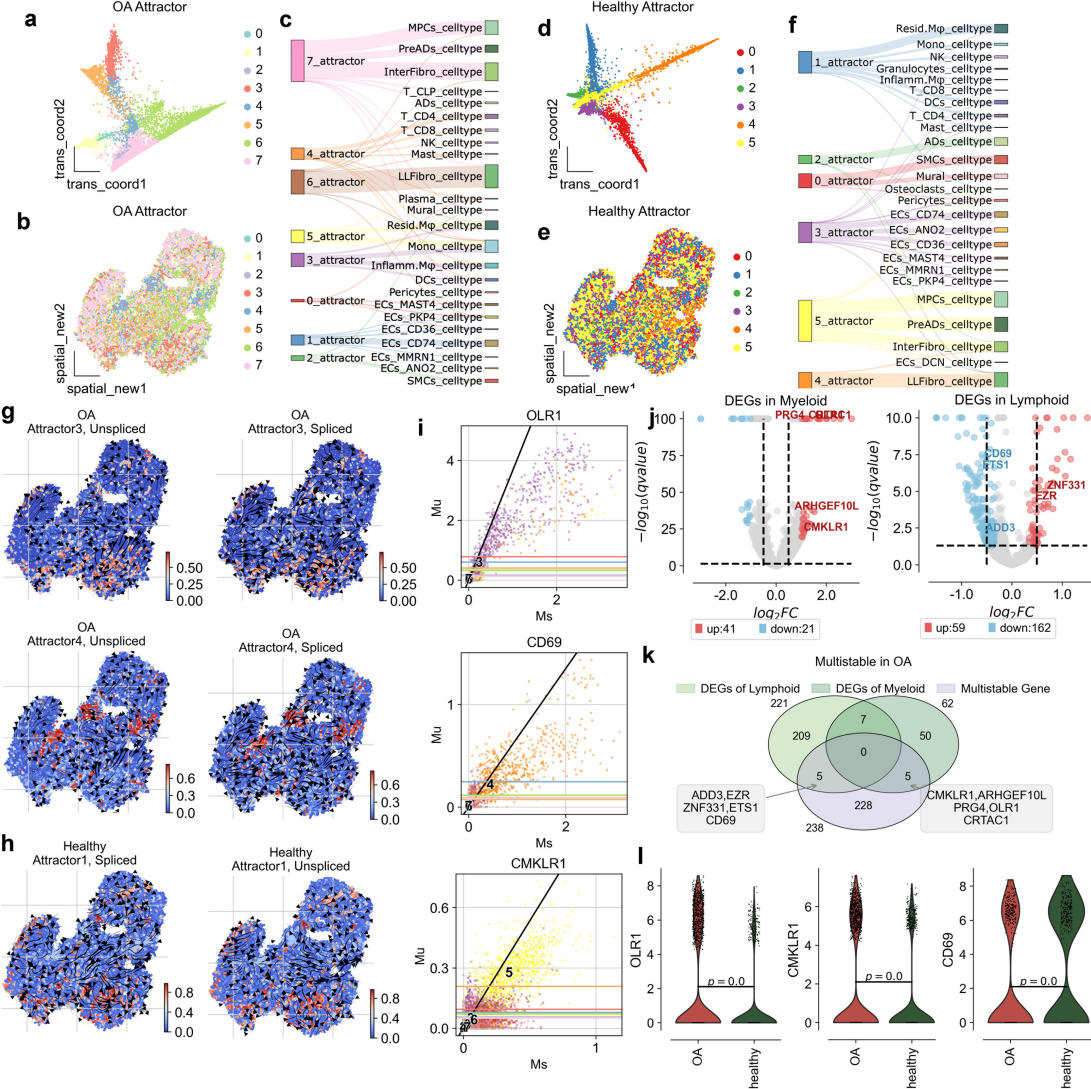
Attractor states and differential gene expression in osteoarthritis (OA) synovium. **(a)** Visualization of OA attractor states in the transition map, showing distinct cellular trajectories. **(b)** Spatial map of synovial tissue, indicating the distribution of attractor states across the tissue in OA. **(c)** Sankey diagram illustrating the relationship between attractor states and cell types, showing the flow through different states. **(d)** Visualization of healthy attractor states in the transition map, highlighting distinct cellular trajectories. **(e)** Spatial map of synovial tissue, indicating the distribution of attractor states across the tissue in healthy conditions. **(f)** Sankey diagram displaying the relationship between attractor states and cell types, illustrating cellular flow through different states. **(g)** Spatial distribution of unspliced and spliced RNA in OA attractor states 3 for Myeloid and 4 for Lymphoid, highlighting areas of high transcriptional activity. **(h)** Spatial distribution of unspliced and spliced RNA in healthy attractor states 1 for Myeloid and 4, highlighting high transcriptional activity. **(i)** Scatter plots of gene expression levels for OLR1, CD69 and CMKLR1, comparing unspliced (Mu) and spliced (Ms) RNA counts in OA conditions. **(j)** Volcano plots of differentially expressed genes (DEGs) in myeloid and lymohoid. **(k)** Venn plot showing the overlap of DEGs in myeloid and lymphoid cells, and genes that are multistable in OA. **(l)** Violin plots showing expression levels of OLR1, CD69, and CMKLR1 in OA and healthy synovium, highlighting significant differences.

**Figure 4:**
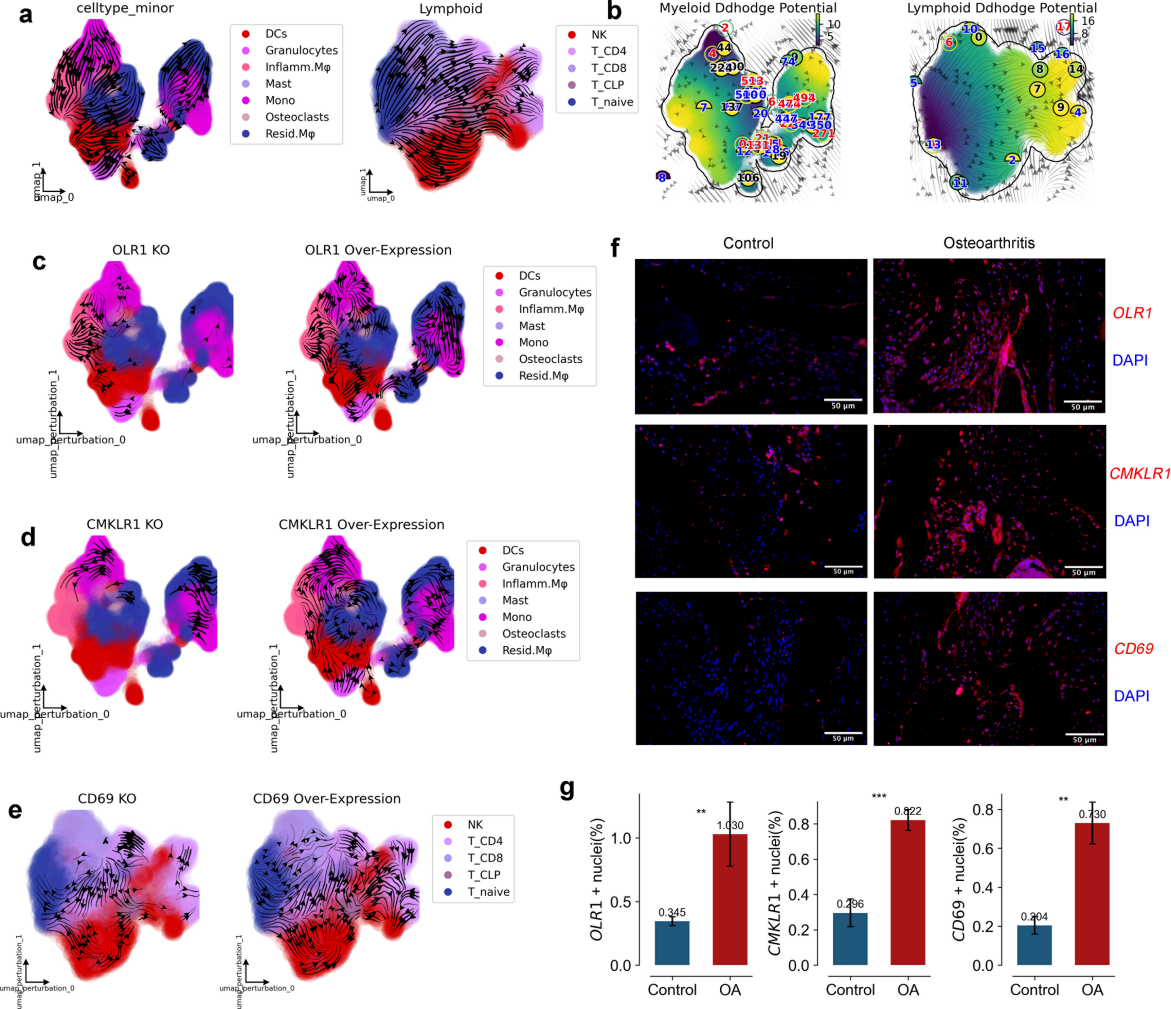
RNA Velocity, Developmental Potential, and Experimental Validation of Key Genes in Myeloid and Lymphoid Cells. **(a)** UMAP graph with RNA velocity streamlines overlain, to depict the flow of cell states over time based on splicing ratios for each gene. Arrowheads depict ‘flow’ of RNA velocities Left panel is Myeloid, right panel is Lymphoid. **(b)** UMAP graph with RNA velocity streamlines showing the Ddhodge potential (In fact, it is the negative of potential here for the purpose to match up with the common usuage of pseudotime so that small values correspond to the progenitor state while large values terminal cell states.). **(c, d)** Streamflow of Myeloid development, presenting profiles in original data, with OLR1, CMKLR1 overexpression, and with OLR1, CMKLR1 knockdown. **(e)** Streamflow of Lymphoid activation, presenting profiles in original data, with CD69 overexpression, and with CD69 knockdown. **(f, g)** Staining **(f)** and quantification **(g)** of OLR1, CMKLR1 and CD69 in human Synovium of Control and Osteoarthritis. (n=3) (*p < 0.05, **p < 0.01, ***p < 0.001).

To further explore the disruption of synovial space homeostasis in osteoarthritis (OA), we used spatial transition tensor (STT), a method that uses messenger RNA splicing and spatial transcriptomes through a multiscale dynamical model to characterize multistability in space (19) to simultaneously construct synovial space homeostasis maps in both OA and Healthy samples (**Figures 3a**, **4a, b**) . In OA, we identified 8 types of attractors (**Figures 3a-c**), while in Healthy samples, 6 types of attractors were identified (**Figures 3d-f**). We found that in OA, Attractor3 is associated with immune myeloid cells, representing the myeloid spatial homeostasis point; Attractor4 is related to lymphocytes, representing the spatial homeostasis point of lymphatic infiltration; and Attractor5 is associated with resident myeloid cells (**Figure 3c**). In Healthy samples, there is no specific attractor related to lymphatic infiltration, with only Attractor1 associated with resident myeloid cells (**Figure 3f**). Through spatial dynamics, we discovered that, compared to Healthy samples, OA exhibits specific spatial homeostasis conditions of immune myeloid and lymphatic infiltration (**Figures 3g, i**).

To uncover the driving genes of spatial homeostasis in lymphatic infiltration and immune myeloid cells in OA, we first calculated the differentially expressed genes in myeloid and lymphoid cells. In myeloid cells, 41 upregulated genes and 21 downregulated genes were identified (**Figure 3j**). For the spatial homeostasis in OA, STT identified a total of 238 homeostasis genes. Among them, ADD3, EZR, ZNF331, ETS1, and CD69 were identified as spatial homeostasis genes for lymphatic infiltration, while CMKLR1, ARHGEF10L, PRG4, OLR1, and CRTAC were identified as driving genes for the spatial homeostasis of immune myeloid cells (**Supplementary Tables 1**, **2**).

In Attractor3, the splicing dynamics slope of OLR1 is smaller than 1, indicating an immature state. A splicing dynamics slope below 1 in our velocity-based analysis suggests a higher proportion of unspliced (newly transcribed) mRNA relative to spliced (mature) mRNA for OLR1 in myeloid cells within this attractor. This “immature” state can be interpreted as active transcription and potentially increasing expression levels of OLR1 in these cells. OLR1 is indeed expressed at higher levels in myeloid cells of OA than in healthy individuals (**Figures 3h, k**), consistent with active gene expression associated with the attractor state. OLR1(also known as Lox1) has been found in past reports to have higher expression on synovium in OA than in Healthy and to be enriched on macrophages (20–22), but the exact mechanism is unclear. We used dynamo to construct a developmental atlas of splicing dynamics of myeloid cells in OA, and we subsequently modeled the effects on the development of the myeloid lineage profile in OA in the case of OLR1 overexpression and OLR1 knockdown, respectively. the developmental profile of Mono to Inflamm.Mφ was inhibited when OLR1 was knocked down, and when OLR1 was overexpressed the developmental profile of Mono to Inflamm.Mφ was inhibited, and when OLR1 was overexpressed the developmental profile of Mono to Inflamm.Mφ was inhibited. developmental lineage was significantly activated when OLR1 was overexpressed, consistent with the upregulation of OLR1 expression in OA (**Figures 4c, d**).

In Attractor4, the splicing dynamics slope of CD69 is less than 1 also, indicating a mature state activation of T cells, spatial homeostasis of lymphatic infiltration exists only in OA(**Figure 3h**). CD69 has been found in past reports to have higher expression on synovium in OA and RA than in Healthy and to be enriched on T cells (23–25), but the exact mechanism is unclear. We used dynamo to construct a developmental atlas of T cell splicing dynamics in OA, and we subsequently modeled the effects on OA T cell lineage development in the case of CD69 overexpression and CD69 knockdown, respectively. the developmental profiles of T naive to T CD4+ and T CD8+ were inhibited when CD69 was knocked down, and when CD69 was overexpressed, the developmental profiles of T naive to T CD4+ and T CD8+ developmental lineages were significantly activated when CD69 was overexpressed, consistent with the upregulation of CD69 expression in OA (**Figure 4e**).

Further, we conducted immunofluorescence verification on synovial tissue and found that OLR1, CD69 and CMKLR1 were all expressed in synovial tissue. Therefore, we also confirmed *in vivo* that OLR1, CD69 and CMKLR1 are key genes in the formation of lymphatic infiltration space in synovial tissue (**Figures 4f, g**). Our study reveals, for the first time, the kinetic characteristics and inflammatory mechanisms of lymphatic infiltration on the spatial surface of osteoarthritis.

### Dysregulated ligand-receptor interactions in osteoarthritis synovial signaling

To elucidate the specific signaling factors involved in the spatial steady state of immune myeloid and lymphatic infiltration in osteoarthritis (OA), we employed a comprehensive multi-step approach. Initially, we utilized CellPhoneDB to computationally analyze interactions among seven distinct cell types in both OA patients and healthy individuals, thus identifying key ligand-receptor pairs critical for cellular communication. Subsequently, we leveraged pyLIGER to construct 20 Gene Modules (GEMs), enabling us to discern the specificity of regulatory modules in signal transduction across diverse cell types.

**Figure 5:**
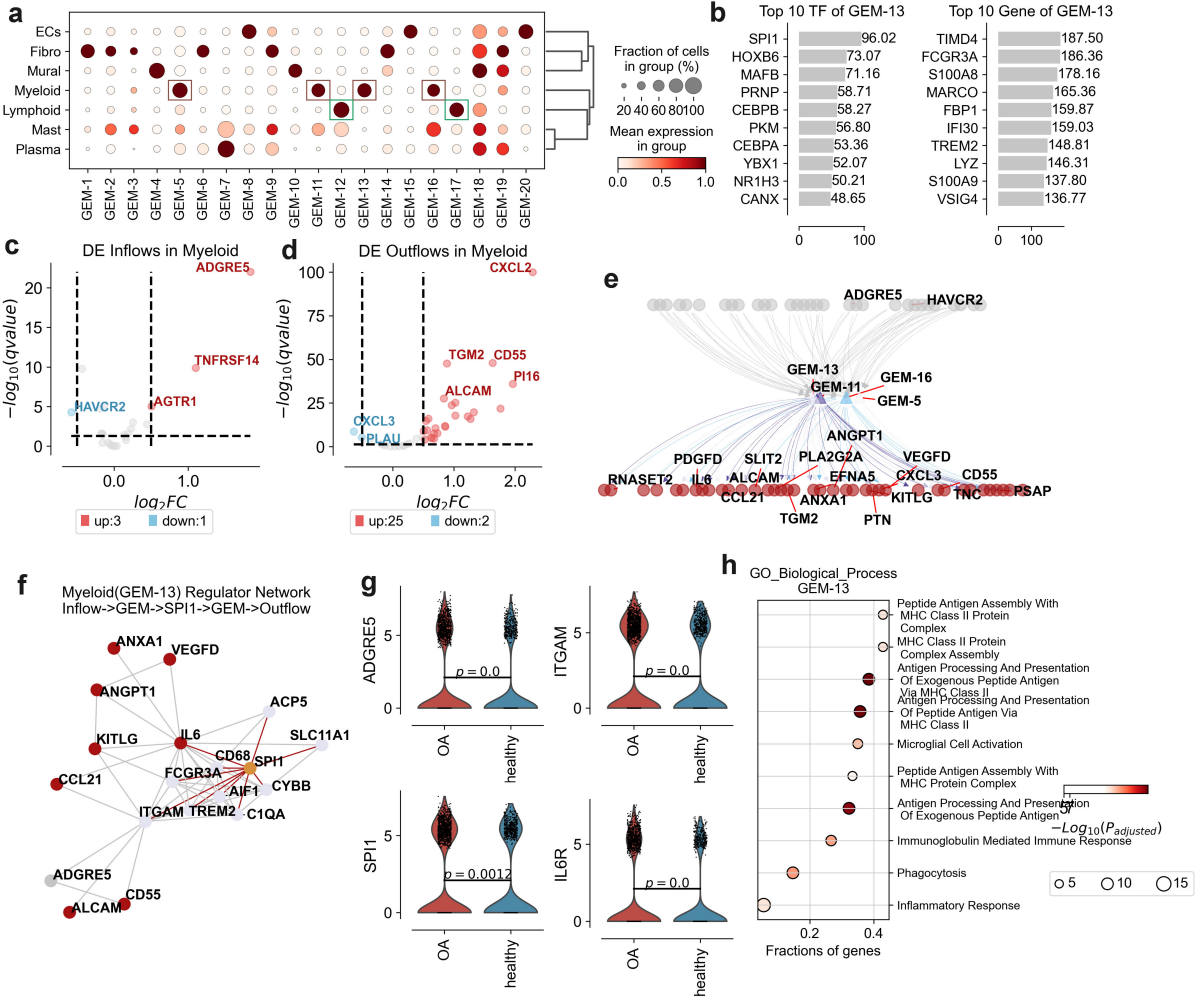
Gene expression modules (GEMs) and transcriptional regulation in osteoarthritis (OA) synovium. **(a)** Dot plot displaying the fraction of cells and mean expression levels of genes in different GEMs across cell types: endothelial cells (ECs), fibroblasts (Fibro), lymphoid cells (Lymphoid), mast cells (Mast), mural cells (Mural), myeloid cells (Myeloid), and plasma cells (Plasma). Dot size indicates cell fraction; color intensity represents mean expression level. **(b)** Top 10 transcription factors (TFs) and genes associated with GEM-13, ranked by significance score. **(c)** Volcano plot of differentially expressed (DE) inflow genes in myeloid cells, comparing OA to healthy synovium. Key genes are labeled, with upregulated genes in red and downregulated in blue. **(d)** Volcano plot of DE outflow genes in myeloid cells, comparing OA to healthy synovium. Key genes are labeled, with upregulated genes in red and downregulated in blue. **(e)** Network diagram illustrating interactions between key genes in GEM-13, GEM-11, GEM-5, and GEM-16, highlighting significant DE genes or DE flows genes. The grey represent inflow signal receptor, red represent outflow ligand. **(f)** Network diagram depicting interactions between key genes and TFs of GEM-13, including inflow receptors and outflow ligands. Red indicates outflow ligands, grey inflow ligands, orange TFs, and other colors represent GEM genes. **(g)** Violin plots showing expression levels of ADGRE5, ITGAM, SPI1, and IL6R between OA and healthy groups. **(h)** Gene Ontology (GO) enrichment analysis for biological processes associated with GEM-13, showing the fractions of genes involved in each process. The size of the dots represents the fraction of genes, while the color intensity indicates the -log10(p-adjusted) value.

Our analysis revealed that GEM-5, GEM-11, GEM-13, and GEM-16 are predominantly associated with myeloid cells, while GEM-12 and GEM-17 are primarily linked to lymphocytes (**Figure 5a**, **Supplementary Figure 4a**). Further investigation identified the Top 10 transcription factors (TFs) for these six GEMs. In myeloid cells, SPI1 emerged as the specific TF for GEM-13. SPI1, also known as PU.1, has been implicated in the regulation of immune response genes, which are crucial in OA due to their role in inflammation and synovial macrophage activity (26). TFEC was identified as the specific TF for GEM-5, and although its direct link to OA is less documented, it is known to influence macrophage differentiation, which plays a role in OA pathogenesis (27). MAF was identified for GEM-11, directly related to granulocyte functions affecting inflammation and tissue remodeling in OA (28). NFKB1 was identified for GEM-16, a pivotal factor in inflammatory response regulation in OA, as NFKB1 is involved in the expression of pro-inflammatory cytokines in osteoarthritic cartilage (29). In lymphocytes, BCL11B was identified as the specific TF for GEM-12. BCL11B is crucial for T-cell development and function, thus impacting immune responses in RA (30). MCTP2 was identified as the specific TF for GEM-17, a module regulating natural killer (NK) cells, which have been implicated in OA due to their role in synovial inflammation (31) (**Figures 5b-d**; **Supplementary Figures 4b, c**).

After we performed Gene Ontology enrichment analysis on each GEM, we found that GEM-13 functionally correlated with MHC Class II Protein Complex Assembly and Peptide Antigen Assembly With MHC Class II Protein Complex function (**Figure 5h**). We concluded that GEM-13 is associated with Myeloid infiltration. Subsequently, we used String-DB to further investigate the upregulated inflow signaling ADGRE5 and upregulated outflow signaling set of Myeloid in relation to TF and Genes in GEM-13. We revealed ADGRE5->ITGAM->SPI1->IL6, ADGRE5->ITGAM->SPI1->IL6->ALCAM, ADGRE5->ITGAM->SPI1->ITGAM->CD55, and others (**Figures 5e-g**). suggests that inflammatory macrophages in osteoarthritis are extensively affected by ADGRE5 activation of their own SPI1 to regulate of inflammation, phagocytosis, and cell signaling.

In addition, the function of GEM-12 was found to correlate with the function of T-cell activation (**Supplementary Figure 4c**), and we found the formation of T-cell aggregation homeostasis to be one of the hallmarks of OA in our previous analysis (**Figure 2g**). We concluded that GEM-12 is associated with T cell activation. Subsequently, we used String-DB to further investigate the relationship between the up-regulated inflow signal IL6R and the up-regulated outflow signal CXCL2 of Lymphoid T cells with TFs and Genes in GEM-13. We revealed the signaling process IL6R->IL7R/PTPRB->BCL11B/STAT4->IL7R->CXCL2 (**Supplementary Figures 4e-g**). It is shown that the activation of T cell homeostasis in osteoarthritis is mediated by IL6R. CXCL2, a chemokine involved in the recruitment of inflammatory cells to the synovial space promote the inflammation (32).

Further, we conducted immunofluorescence verification on synovial tissue and found that OLR1, CD69 and CMKLR1 were all expressed in synovial tissue. Therefore, we also confirmed *in vivo* that OLR1, CD69 and CMKLR1 are key genes in the formation of lymphatic infiltration space in synovial tissue.

## Discussion

Understanding the spatial and signaling dynamics, as well as the splicing kinetics of immune infiltration in osteoarthritis (OA) synovium, sheds light on the complex pathogenesis of this debilitating joint disorder. The infiltration of immune cells, particularly myeloid and lymphoid cells, within the synovial membrane contributes to the chronic inflammatory environment characteristic of OA. Despite advances in our understanding of OA at the cellular level, spatial and transcriptional characterizations remain incomplete. In this study, we employ cutting-edge spatial transition tensor and intercellular flow analyses to delineate the signals regulating immune cell infiltration in OA synovium, providing novel insights into the spatial dynamics and signaling mechanisms involved.

Our study successfully identifies distinct spatial domains within the synovial membrane, highlighting the differential distribution and transcriptional states of immune myeloid and lymphoid cells in OA compared to healthy controls. The spatial attractors identified in OA synovium underscore the unique spatial homeostasis conditions of immune infiltration, driven by key transcription factors and differentially expressed genes. Notably, the genes OLR1, CD69, and CMKLR1 emerge as critical regulators of spatial homeostasis in OA, with their expression patterns reflecting the complex interplay between immune infiltration and tissue remodeling. Consistent with our computational predictions, we have now experimentally validated the expression of OLR1, CD69, and CMKLR1 in OA synovial tissue using immunofluorescence (**Figure 4g**), further supporting their role in OA pathogenesis. Furthermore, we identified key transcription factors (SPI1, MAF, NFKB1, BCL11B, MCTP2) orchestrating signaling networks within myeloid and lymphoid cells. These findings highlight potential therapeutic avenues in OA. Specifically, OLR1 and CMKLR1 emerge as promising targets for directly modulating pathogenic immune responses within the synovium (22, 33). Targeting these receptors could directly reduce myeloid cell infiltration and inflammation. Concurrently, modulating the identified transcription factors offers a strategy to broadly dampen inflammatory signaling cascades within specific immune cell populations in the OA joint. These targeted approaches, focused on spatial and signaling dysregulation, hold the potential to complement existing OA therapies and guide the development of novel, more effective treatments aimed at restoring immune homeostasis and alleviating the inflammatory burden in OA. Future research should prioritize preclinical validation of these targets to accelerate the translation of our findings into clinical applications.

The spatial specificity of myeloid cell infiltration, particularly within the synovial lining and sub-synovial layers, emphasizes the localized inflammatory responses that contribute to OA pathogenesis. These spatial disruptions may serve as potential biomarkers for disease severity or progression. For instance, the enrichment of OLR1 and CMKLR1 in specific synovial regions could be leveraged to develop imaging-based diagnostic tools or targeted therapies that modulate immune cell infiltration in these areas.

Our analysis of signaling dynamics reveals significant abnormalities in the spatial signaling landscape of OA synovium. Through the construction of gene modules and identification of ligand-receptor interactions, we uncover key regulatory modules associated with myeloid and lymphoid cells. These findings highlight the pivotal role of transcription factors such as SPI1, MAF, and NFKB1 in modulating inflammatory processes and tissue degradation in OA. These results are consistent with previous studies demonstrating the involvement of NF-κB signaling in OA inflammation (34, 35), but our work extends these findings by linking specific transcription factors to spatial signaling networks in the synovium. The identification of these signaling networks offers potential therapeutic targets for modulating immune cell signaling and mitigating the inflammatory milieu in OA joints. For example, targeting SPI1 or MAF could disrupt pro-inflammatory signaling cascades, while modulating ligand-receptor interactions identified in our study may restore immune homeostasis. These approaches could complement existing therapies, such as anti-inflammatory drugs or biologics, by addressing the spatial and signaling dysregulation underlying OA pathogenesis.

Our findings build on and expand the growing body of literature using spatial transcriptomics and single-cell technologies to study OA. While previous studies have characterized immune cell populations and their transcriptional profiles in OA synovium (22, 33), our study is among the first to integrate spatial dynamics and signaling networks to provide a comprehensive atlas of immune infiltration in OA. This approach not only confirms previously identified pathways but also reveals novel spatial and transcriptional regulators, such as OLR1 and CD69, that may play critical roles in OA pathogenesis.

This study has several inherent limitations that warrant acknowledgment. Firstly, the sample size, while sufficient to reveal key trends, remains modest. Larger cohorts are needed to enhance statistical power and fully capture the heterogeneity of OA. Secondly, our reliance on publicly available data introduces potential limitations from the original datasets’ quality and design, though we attempted to mitigate batch effects computationally. Furthermore, despite immunofluorescence validation, the absence of functional assays directly testing the predicted roles of identified genes and pathways remains a key limitation. Future research should prioritize functional validation and explore these targets in larger, more diverse patient cohorts to solidify our computational predictions and advance translational applications.

In conclusion, our study provides a comprehensive atlas of the spatial and signaling dynamics of immune infiltration in OA synovium, advancing our understanding of OA pathogenesis. The identification of key transcriptional regulators and signaling networks offers valuable insights into potential therapeutic strategies aimed at targeting immune cell signaling to alleviate the inflammatory burden in OA. These findings pave the way for future research focused on the development of targeted therapies that modulate immune infiltration and restore joint homeostasis in OA patients. Future studies should validate these findings in larger cohorts and explore the translational potential of identified biomarkers and therapeutic targets in preclinical and clinical settings.

## Method

### Immunofluorescence analysis

The process of bone tissue preparation involves several crucial steps: decalcification, dehydration, rendering it transparent, wax embedding, slicing, baking, and dewaxing. For antigen retrieval, an enzymatic digestion method was employed, utilizing a bone tissue antigen repair solution sourced from Solarbio, located in Beijing, China. Subsequently, the bone tissue underwent blocking with sheep serum (also from Solarbio) at a temperature of 25°C for a duration of 30 minutes.Primary antibody reactions were conducted using specific antibodies: CMKLR1 antibody [EPR26501-70], diluted 1:50 (ab306554; Abcam), CD69 antibody [EPR25398-81], diluted 1:50 (ab307081; Abcam), and OLR1 antibody, diluted 1:50 (11837-1-AP; Proteintech). These antibodies were incubated overnight at a temperature of 4°C.For secondary antibody reactions, Goat Anti-Rabbit IgG antibodies were used, diluted 1:1000 (Alexa Fluor^®^488, ab150077; Alexa Fluor^®^647, ab150079; both from Abcam). This incubation step was carried out at 37°C for a period of 2 hours. Nuclear labeling was achieved using DAPI.Finally, the prepared slides were mounted with an anti-fluorescent quencher, and images were captured using an Olympus BX53 microscope for further analysis.

### scRNA-seq acquisition and preprocessing

Single-cell RNA sequencing (scRNA-seq) of osteoarthritis (OA) and healthy synovial tissue were obtained from the Gene Expression Omnibus (GEO) database (accession numbers: GSE216651 (33), GSE152805 (22)). All patients provided a written informed consent. Clinical characteristics of the patients are collected as **Table 1**. All single-cell raw reads are preprocessed through Cellranger 7.0.0 (17), featuring re-mapping against the reference human genome hg38 (36) for acquisition of count expression matrices. Furthermore, Velocyto (37) is employed to extract unspliced and spliced expression matrices from the bam files of every single-cell sequencing sample.

We apply the `omicverse.pp.qc` function from the Omicverse (38) package sequentially to each sample for quality control. The basic cellular screening criteria are automatically calculated using Mads for every cell’s gene counts threshold, with mitochondrial genes ration below 20%. Double-cell filtering is executed using scrublet (39) for quality control. Furthermore, we use `omicverse.pp.preprocess` on the integrated data to standardize and log-transform it. We estimate highly variable genes employing Pearson residuals method. The parameters are set as mode=“shiftlog|pearson”, target_sum=50*1e4, and n_HVGs=3000.

### Spatial RNA-seq acquisition and preprocessing

Spatial transcriptomics data for synovial tissue of rheumatoid arthritis have been deposited at ImmPort (https://www.immport.org) under study accession SDY2213. All spatial raw reads are preprocessed through SpaceRanger 3.0.0, featuring re-mapping against the reference human genome hg38 for acquisition of count expression matrices.

We apply the `scanpy. pp.calculate_qc_metrics` from the Scanpy package to calculate the metric of each spots and filtered reads lower than 100 spots. Furthermore, we use `scanpy.pp.preprocess` and `scanpy.pp.log1p` on the integrated data to standardize and log-transform it. We then estimate highly variable genes employing Prost (40) method of `omicverse.space.SVG`. The parameters are set as mode=“Prost”, and n_HVGs=3000.

### Batch correction and annotation

For batch effect correction, to integrate 9 synovial samples, we utilized scVI (41) implemented via `omicverse.single.batch_correction` with the following parameters: `methods=‘scVI’`, `n_layers=2`, `n_latent=30`, and `gene_likelihood=“nb”`. We chose `n_layers=2` and `n_latent=30` as they are established parameters for effective scVI-based batch correction in scRNA-seq analyses, balancing model complexity and computational efficiency. The `gene_likelihood` parameter was set to `”nb”` to appropriately model RNA-seq count data using a Negative Binomial distribution.

Following this, we used the Leiden (42) algorithm based on the low-dimensional vector X_scVI output by scVI to perform unsupervised automatic clustering of integrated cells with a resolution parameter set at 1.This resolution was empirically determined to provide biologically meaningful major cell groups, balancing cluster granularity and interpretability based on marker gene analysis and cell type coherence. For refined sub-clustering within specific cell types (Fibroblasts, Myeloid cells, Lymphoid cells, and Vascular cells), Leiden clustering was re-applied, and a resolution parameter of `1` was chosen after testing resolutions from `0.5` to `2`. A resolution of `1` for sub-clustering provided a detailed yet interpretable sub-structure within these major cell populations, avoiding over-segmentation and maintaining biological relevance of sub-clusters based on examination of cluster-specific marker genes using differential gene expression analysis (`scanpy.tl.rank_genes_groups`).

We then utilized `omicverse.single.GPT4Celltype` to automatically annotate cell types for every cluster through iterative refinement over 5 rounds (43). We then applied COSG (44) to determine marker genes for each cluster. Based on this analysis, we identified Myeloid marked by CD163, Fibroblast by COL1A2, Endothelial by EPCAM1, SMCs by ACTA2, and Lymphoid by IL7R, distinguishing five major types of OA. Further subdivision was achieved by considering common marker genes.

For cell type-aware annotation refinement, we employed scANVI (45), building upon the pre-trained scVI model using `scvi.model.SCANVI.from_scvi_model`. The scANVI model was trained for `25 epochs` to fine-tune cell type classification within the batch-corrected latent space. The epoch number was set through empirical testing to achieve robust annotation while avoiding overfitting.

### Cell location of scRNA-seq

To obtain the spatial location distributions of OA and Healthy synovial membrane separately, we utilized spatrio (46) to integrate OA scRNA-seq data with synovial membrane ST data. This process involved optimal transport metrics to determine the optimal spot position for each cell across these datasets.

Furthermore, using COSG, we computed marker genes for each spatial domain within the ST data. These marker gene expressions were then used to compare and confirm the spatial distribution patterns of OA versus Control synovial membrane.

To obtain the spatial location of each single cell, we first employed the Tangram (47) algorithm from `omicverse.space.tangram`, using the integrated single-cell data as a reference. This allowed us to determine the cell proportions for every spot in the spatial transcriptome. Subsequently, we utilized spatrio (46) to localize individual cells within their respective positions on the spatial transcriptomic map. Furthermore, we using `omicverse.pp.cosg`’s algorithm COSG (44), we computed marker genes for each spatial domain within the ST data. These marker gene expressions were then used to compare and confirm the spatial distribution patterns of OA and Healthy synovial membrane cells.

### Spatial transition tensor analysis

We implemented the STT (48), as described by Svensson et al, was applied to characterize spatial stability and multistability in the synovial membrane, analysis using the `omicverse.space.STT` function, and trained the model using `STT_obj.train()` with the following parameters: `n_states = 8`, `n_iter = 15`, `weight_connectivities = 0.5`, `n_neighbors = 50`, `thresh_ms_gene = 0.2`, and `spa_weight =0.3`. The number of attractors, `n_states = 8`, was initialized based on the number of clusters identified by Leiden clustering in the `STT_obj.stage_estimate()` step, serving as an initial estimate for stable spatial states. We set `n_iter = 15` and `n_neighbors = 50` to ensure sufficient iterations for robust tensor learning and to consider a sufficiently broad local neighborhood respectively. `weight_connectivities = 0.5` was used to equally weigh spatial proximity and gene expression connectivity in the tensor construction, balancing spatial and transcriptional dynamics. We applied a threshold `thresh_ms_gene = 0.2` to filter genes for splicing dynamics analysis, focusing on genes exhibiting active transcriptional changes as reflected by sufficient unspliced and spliced mRNA ratios. The spatial weight, `spa_weight = 0.3`, assigned a moderate weight to spatial information relative to velocity dynamics in defining spatial attractors, reflecting the importance of spatial context in tissue organization without overpowering the intrinsic transcriptional dynamics captured by RNA velocity. The terminal stages of cellular spatiotemporal homeostasis were identified using CellRank2 (49), and the transition probabilities for each terminal stage were computed.

### Differential expression analysis

Differentially expressed genes (DEGs) between OA and healthy groups were identified using the `omicverse.bulk.pyDEG` function with the t-test. All basemean of DEGs were filtered by 0.5 and considered significant if they had an adjusted p-value < 0.05 and a log2 fold change > 0.25. The DEGs were further intersected with genes identified by the spatial transition tensor (STT) algorithm to determine those associated with spatial homeostasis disruption.

### Visualization and statistical analysis

Data visualization was conducted using Matplotlib and Seaborn packages in Python. Violin plots, dot plots, and heatmaps were used to display gene expression and cell type proportions. Statistical analyses were performed using Python, with p-values adjusted for multiple testing using the Benjamini-Hochberg method. Log2 fold changes were calculated to quantify differences in cell type proportions and gene expression levels between conditions. For quantitative data, the Kolmogorov-Smirnov test (α = 0.05) was performed to assess normality. If the data followed a normal distribution, Levene's test (α = 0.05) was subsequently applied to evaluate homogeneity of variances between groups. Data conforming to both normality and homogeneity of variance assumptions were expressed as mean ± standard deviation (SD). Comparisons between two groups were analyzed using a two-tailed unpaired Student's t-test. For data violating normality or variance homogeneity assumptions, non-parametric tests (e.g., Mann-Whitney U test) were employed.

### Spatial mapping and domain identification

Spatial transcriptomics data were processed using the STAGATE algorithm^26^ to identify spatial domains within the synovial tissue. Spatial clusters were annotated based on hematoxylin and eosin (HE) staining and the expression of region-specific markers. To assign spatial coordinates to single cells, we employed a deconvolution approach combined with optimal transport theory.

### Cell-cell communication analysis

We used the COMMOT algorithm to infer cell-cell communication (CCC) pathways in the spatial transcriptomics data (50). COMMOT considers spatial distances and the competition between different ligand-receptor species to identify spatially constrained CCC pathways. The inferred ligand-receptor interactions were visualized using network diagrams.

### Signaling analysis

FlowSig was used to analyze intracellular signaling pathways driven by the identified CCC pathways (51). FlowSig employs graphical causal modeling and conditional independence to infer communication-driven intercellular flows. This analysis allowed us to identify key signaling molecules and pathways involved in myeloid and lymphoid cell infiltration in OA. The interaction of genes were calculated by String-DB (52).

### Differential expression analysis

Differential expression (DE) analysis was performed using the Scanpy’s rank_genes_groups function, which implements the Wilcoxon rank-sum test. DE genes were identified between OA and healthy synovium, with significance thresholds set at adjusted p-value < 0.05 and log2 fold change (log2FC) > 0.25.

### Gene ontology enrichment analysis

Gene ontology (GO) enrichment analysis was conducted using the GSEApy tool (53). Enriched GO terms were identified for gene expression modules (GEMs) associated with spatial attractors and DE genes, highlighting biological processes relevant to OA pathogenesis.

## Data Availability

Publicly available datasets were analyzed in this study. This data can be found here: scRNA-seq Acquisition and PreprocessingSingle-cell RNA sequencing (scRNA-seq) of osteoarthritis (OA) and healthy synovial tissue were obtained from the Gene Expression Omnibus (GEO) database [accession numbers: GSE216651 (33), GSE152805 (22)].
