# Supplementary material for: Unraveling the spatial and signaling dynamics and splicing kinetics of immune infiltration in osteoarthritis synovium
Source: Front Immunol. 2025 Mar 13;16:1521038. doi: 10.3389/fimmu.2025.1521038 (PMC11966058; doi:10.3389/fimmu.2025.1521038)
Supplement: Supplementary file 1 [file DataSheet1.pdf]

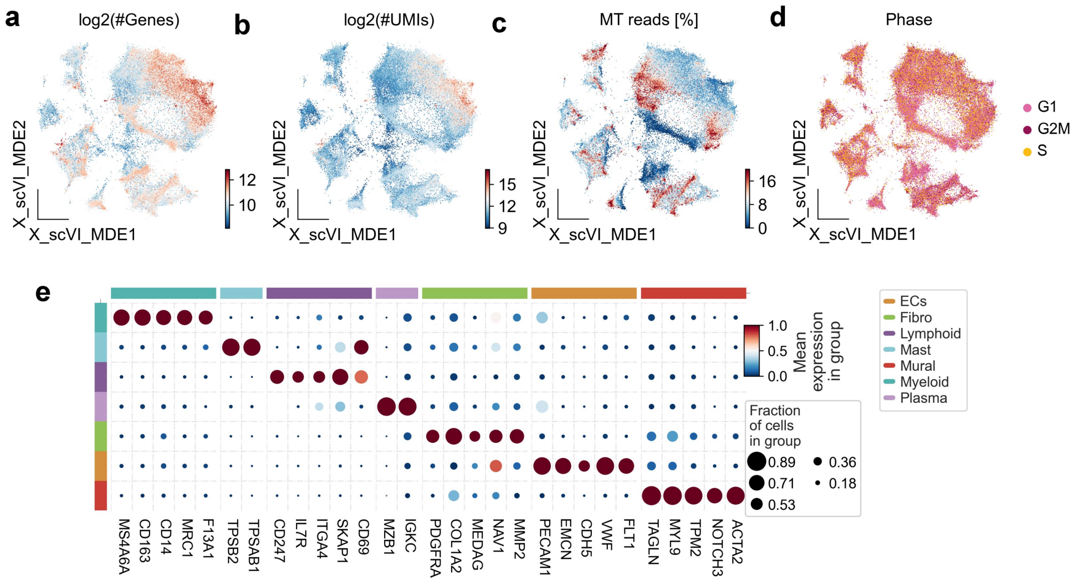

Supplement Figure 1 Analysis of single-cell transcriptomic data.

(a-d) Dimensionality reduction visualization using the scANVI algorithm, showing the number of genes (log2(#Genes)), number of UMIs (log2(#UMIs)), percentage of mitochondrial reads (MT reads [%]), and phase of cell respectively.

(e) Dot plot illustrating the expression of specific genes within each cell group. Dot size represents the fraction of cells in the group, and color intensity indicates the mean expression level of the genes.

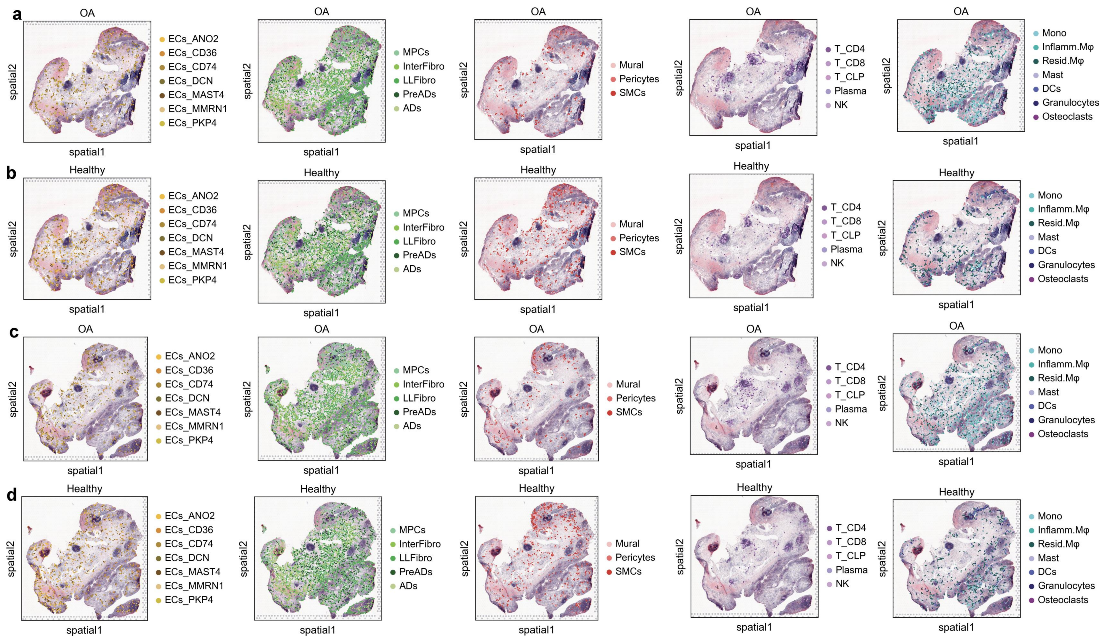

Supplement Figure 2 Spatial distribution of cell types in osteoarthritic (OA) and healthy tissues.

(a-d) Visualization of spatial transcriptomics data illustrating the distribution of various cell types across OA and healthy samples. Cell types include endothelial cells (ECs), mesenchymal progenitor cells (MPCs), mural cells, immune cells, and others, as indicated by the color-coded legends. Each panel represents a different cell type category: (a) Endothelial cells, (b) Fibroblasts and adipocytes, (c) Mural cells, and (d) Immune cells. Spatial coordinates are denoted by spatial1 and spatial2 axes.

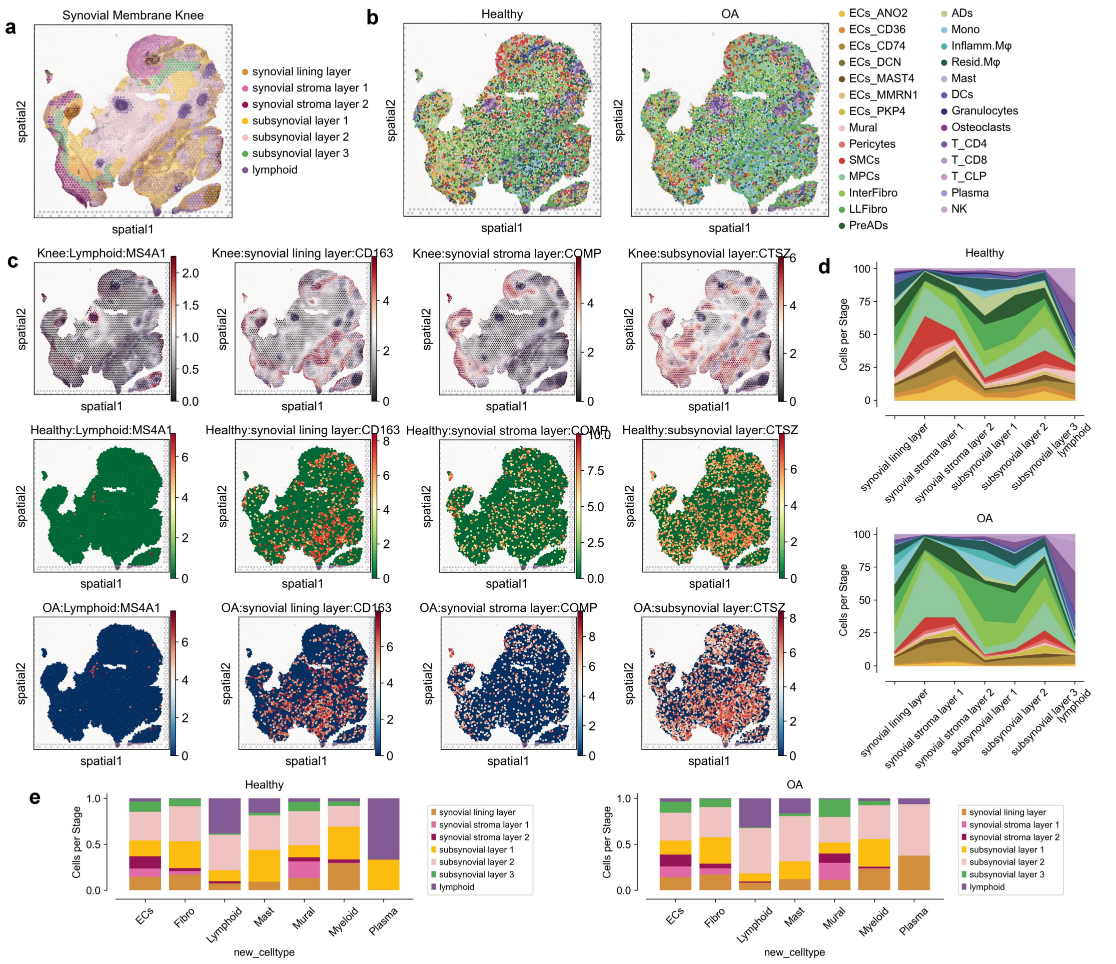

Supplement Figure 3 **Spatial and cellular characterization of knee synovial membrane in healthy and osteoarthritic (OA) conditions.**

(a) Annotated spatial map of the knee synovial membrane, highlighting different anatomical layers such as synovial lining, synovial stroma, sub-synovial layers, and lymphoid regions.

(b) Spatial distribution of various cell types in healthy and OA samples. The legend indicates cell types, including endothelial cells (ECs), fibroblasts (Fibro), immune cells, and others.

(c) Expression patterns of selected marker genes (MS4A1, CD163, COMP, CTSZ) in specific layers of the synovial membrane across healthy and OA conditions.

(d) Area plots showing the proportion of cells per anatomical layer for healthy and OA samples.

(e) Bar plots representing the distribution of cell types across different anatomical layers in healthy and OA conditions. The legend denotes the anatomical layers.

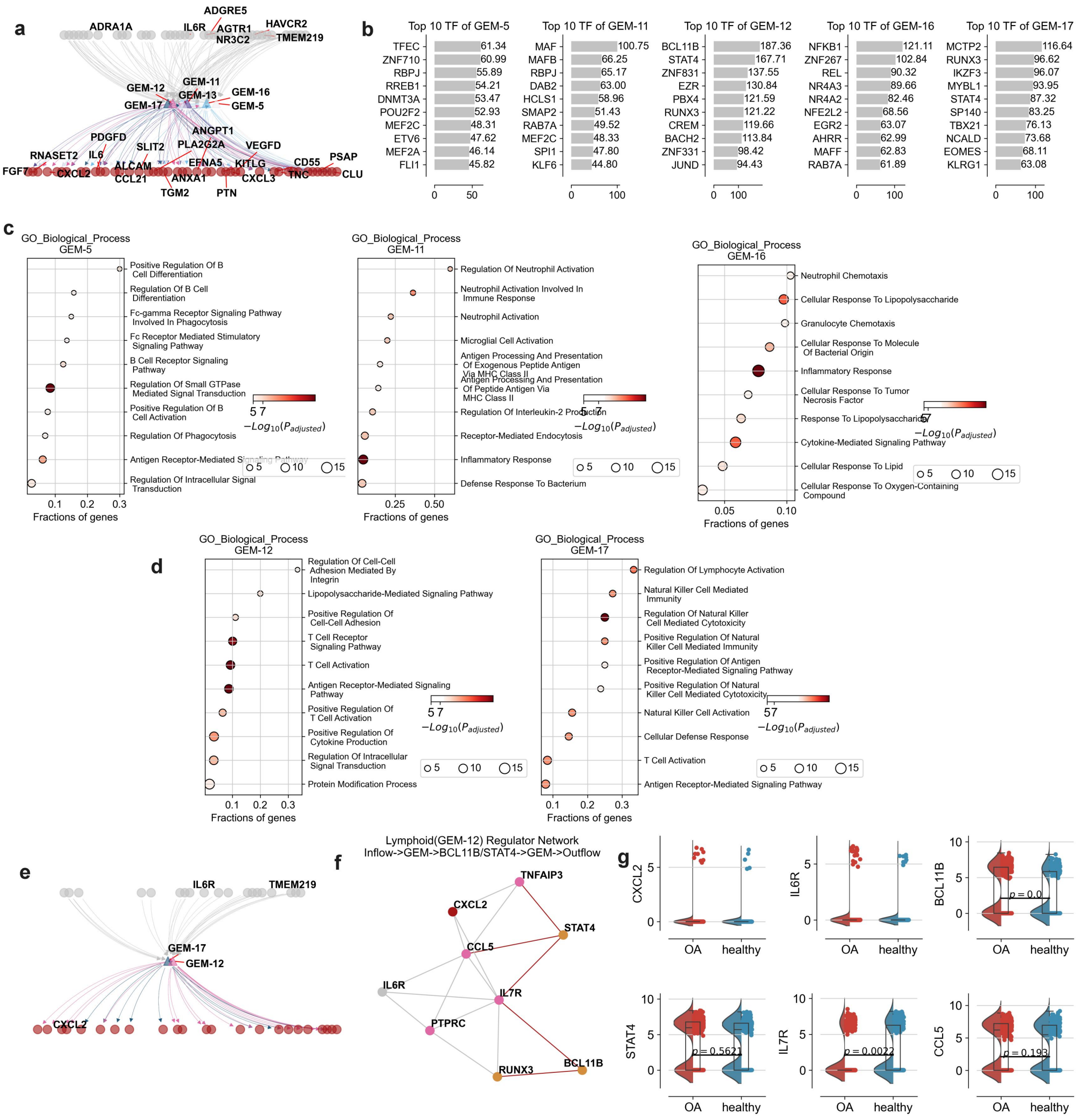

Supplement Figure 4 **Gene expression modules and functional enrichment in osteoarthritic (OA) and healthy conditions.**

(a) Network diagram showing interactions among key genes within identified gene expression modules (GEMs), including GEM-5, GEM-11, GEM-12, and GEM-16.

(b) Top transcription factors (TFs) for each GEM, ranked by their regulatory influence.

(c) Gene Ontology (GO) biological process enrichment for GEM-5, GEM-11, GEM-16 of Myeloid, highlighting significant pathways and processes. Dot size represents the fraction of genes involved, and color intensity indicates the level of significance (-Log10(p-adjusted)).

(d) Gene Ontology (GO) biological process enrichment for GEM-12, GEM-17 of Lymphoid, highlighting significant pathways and processes. Dot size represents the fraction of genes involved, and color intensity indicates the level of significance (-Log10(p-adjusted)).

(e) Network diagram illustrating the interactions between key genes in GEM-12, and GEM-17 and GEM-16, highlighting significant DE genes and their connections.

(f) Network diagram illustrating the interactions between key genes and TF of GEM-12 inflow receptor and outflow ligand, reds represent outflow ligand, grey represent inflow ligand, orange represent TF, and other colors represent the gene in GEM.

(g) Violin plots comparing expression levels of selected genes (CXCL2, IL6R, BCL11B, STAT4, IL7R and CCL5) between OA and healthy samples, illustrating differential expression patterns.
